# Bilosomes as Nanoplatform for Oral Delivery and Modulated In Vivo Antimicrobial Activity of Lycopene

**DOI:** 10.3390/ph15091043

**Published:** 2022-08-24

**Authors:** Reem Binsuwaidan, Amal A. Sultan, Walaa A. Negm, Nashwah G. M. Attallah, Moneerah J. Alqahtani, Ismail A. Hussein, Moataz A. Shaldam, Suzy A. El-Sherbeni, Engy Elekhnawy

**Affiliations:** 1 Department of Pharmaceutical Science, College of Pharmacy, Princess Nourah bint Abdulrahman University, P.O. Box 84428, Riyadh 11671, Saudi Arabia; 2 Department of Pharmaceutical Technology, College of Pharmacy, Tanta University, Tanta 31527, Egypt; 3 Department of Pharmacognosy, Faculty of Pharmacy, Tanta University, Tanta 31527, Egypt; 4 Department of Pharmacognosy, College of Pharmacy, King Saud University, P.O. Box 2457, Riyadh 11451, Saudi Arabia; 5 Department of Pharmacognosy and Medicinal Plants, Faculty of Pharmacy (Boys), Al-Azhar University, Cairo 11884, Egypt; 6 Department of Pharmaceutical Chemistry, Faculty of Pharmacy, Kafrelsheikh University, Kafr El-Sheikh 33516, Egypt; 7 Department of Pharmaceutical Microbiology, Faculty of Pharmacy, Tanta University, Tanta 31527, Egypt

**Keywords:** bilosomes, entrapment efficiency, controlled release, Higuchi kinetics, *Klebsiella pneumoniae*, lung infection

## Abstract

Owing to the disseminating resistance among pathogenic bacteria, especially *Klebsiella pneumoniae*, there is a high need for alternate compounds with antibacterial activity. Herein, lycopene was isolated from *Lycopersicon esculentum* L. Molecular docking approach was employed to explore lycopene binding affinity to selected vital proteins of *K. pneumoniae* with the binding mechanisms being investigated. This proposed a promising antibacterial activity of lycopene. However, the pharmacological use of lycopene is hampered by its poor solubility and limited oral bioavailability. Accordingly, bilosomes were fabricated for oral lycopene delivery. The computed entrapment efficiency, mean vesicular size, and zeta potential values for the optimized formulation were 93.2 ± 0.6%, 485.8 ± 35.3 nm, and −38.3 ± 4, respectively. In vitro drug release studies revealed controlled lycopene release from constructed bilosomes, with the drug liberation being based on the Higuchi kinetics model. Transmission electron microscopic evaluation of bilosomes revealed spherical nanovesicles free from aggregates. Moreover, the in vitro and in vivo antibacterial activity of lycopene and its constructed formulations against multidrug-resistant *K. pneumoniae* isolates were explored. The optimized bilosomes exhibited the lowest minimum inhibitory concentrations ranging from 8 to 32 µg/mL. In addition, scanning electron microscopy revealed remarkable deformation and lysis of the bilosomes-treated bacterial cells. Regarding in vivo investigation, a lung infection model in mice was employed. The tested bilosomes reduced the inflammation and congestion in the treated mice’s lung tissues, resulting in normal-sized bronchioles and alveoli with very few congested vessels. In addition, it resulted in a significant reduction in pulmonary fibrosis. In conclusion, this study investigated the potential activity of the naturally isolated lycopene in controlling infections triggered by multidrug-resistant *K. pneumoniae* isolates. Furthermore, it introduced bilosomes as a promising biocompatible nanocarrier for modulation of oral lycopene delivery and in vivo antimicrobial activity.

## 1. Introduction

*Klebsiella pneumoniae*, Gram-negative bacteria from the family *Enterobacteriaceae*, is one of the gastrointestinal microbiota of healthy individuals, and it is a prevalent opportunistic nosocomial pathogen [1]. It causes extra-intestinal infections such as pneumonia, surgical wound infections, urinary tract infections, endocarditis, and septicemia. Together with its high prevalence, it is acquiring resistance to multiple antimicrobials, increasing morbidity and mortality rates [2]. Thus, it is vital to find new therapeutic alternatives to treat the infections caused by this pathogen, such as naturally derived products.

Lycopene is a natural polyene hydrocarbon with an acyclic structure and 11 linearly conjugated double bonds that gives the bright red color to vegetables, fruits, flowers, and some microbes, particularly photosynthetic one. Tomatoes are humans’ primary dietary lycopene sources [3,4,5]. The antioxidant potential of tomatoes and tomato products is due mainly to lycopene, which plays an essential role in human health. Lycopene has been shown to have various beneficial therapeutic impacts as anticancer, antioxidant, cardioprotective, neuroprotective, anti-inflammatory, antiplatelet aggregative, and antihypertensive effects [6].

Unfortunately, the potential pharmacological use of lycopene is limited due to its poor solubility and extremely low oral bioavailability [7]. This dictates the development of an appropriate delivery system for improving lycopene bioavailability following oral administration.

**Figure 1:**
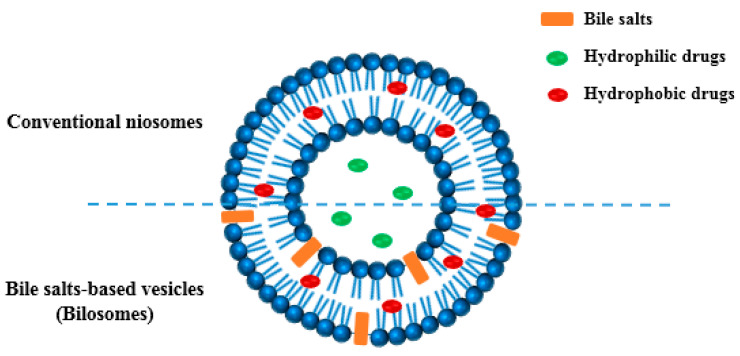
Schematic representation of conventional niosomes and bilosomes as vesicular carriers for both hydrophilic and hydrophobic drugs.

Lipid-based vesicular systems, including liposomes and conventional niosomes, have been adopted for oral delivery of hydrophilic and hydrophobic drugs. However, these systems suffer from limited gastrointestinal stability, payload leakage, and scaling-up problems [8,9]. Bilosomes, bile-salts-incorporating vesicles, are emerging as a colloidal carrier for enhancing the oral delivery of encapsulated drugs. Conacher and co-workers were the first to describe such a system, with subsequent research approaches being conducted over the last decade demonstrating bilosomes potential to improve in vivo drug performance after oral administration [10,11]. Figure 1 presents a schematic illustration of conventional niosomes and bilosomes as vesicular carriers for both hydrophilic and hydrophobic drug moieties. Incorporating bile salts into niosomal vesicles offers greater gastrointestinal stability compared to conventional lipid-based systems [12,13].

Moreover, bile salts may act as intestinal permeation enhancers triggering superior oral drug bioavailability after bilosomal encapsulation [14]. Non-ionic surfactants, including span series, are widely used to formulate bilosomes in combination with cholesterol and bile salts [15]. These components utilized for bilosomes construction are generally considered safe and have been reported to be completely biocompatible and biodegradable [16,17]. These synthetic nano-sized vesicular carriers could provide a platform technology for improving the oral delivery of a wide range of drugs. This was considered regarding the potential of vesicular nanostructures to encapsulate both hydrophilic and hydrophobic agents in the aqueous core and phospholipid bilayer, respectively [17,18].

Accordingly, the current study aimed mainly to find a novel therapy for infections caused by multidrug-resistant *K. pneumoniae* using molecular docking studies as well as in vitro and in vivo investigations. Furthermore, we aimed to tailor a suitable nano-vesicular carrier for its oral delivery. Bilosomal encapsulation of lycopene revealed a promising in vitro activity against *K. pneumoniae*, with the in vivo performance substantially improved compared to the unprocessed form.

## 2. Results

### 2.1. Phytochemical Investigation

**Figure 2:**
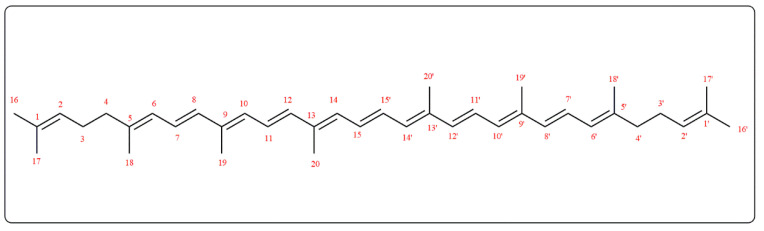
Chemical structure of lycopene.

Lycopene isolated from *Lycopersicon esculentum* L. ESI-MS, ^1^H, and ^13^C-NMR data were compared to those described in the literature [3,19]. Lycopene ESI-MS *m*/*z* was 535.9 [M-H]^−^ with a molecular formula C_40_H_56_. Figure 2 presented the chemical structure of lycopene, while ^1^H-NMR (CDCl_3_, 400 MHz) and DEPT ^13^C-NMR (CDCl_3_, 100 MHz) results are as the following: ^1^H NMR δ: 5.18 (2H, bs, H-2,2′), 2.06 (8H, bs, H-3,3′,4,4′), 5.95 (2H, d, *J* = 11.4, H-6,6′), 6.1–7.1 (14H, m, H-7,7′,8,8′,10,10′,11,11′,12,12′,14,14′,15,15′), 1.7 (6H, s, H-16,16′), 1.6 (6H, s, H-17,17′), 1.91 (6H, s, H-18,18′), 1.97 (12H, s, H-19,19′,20,20′).

DEPT ^13^C-NMR δ: 139.6 (2C, C-1,1′), 137.6 (2CH, C-2,2′), 136.6 (2C, C-3,3′), 136.4 (2C, C-4,4′), 135.9 (2CH, C-5,5′), 132.4 (2CH, C-6,6′), 131.9 (2C, C-7,7′), 131.8 (2CH, C-8,8′), 130.2 (2CH, C-9,9′), 125.7 (2CH, C-10,10′), 124.4 (2CH, C-11,11′), 124.2 (2CH, C-12,12′), 123.9 (2CH, C-13,13′), 40.5 (2CH_2_, C-14,14′), 27.2 (2CH_2_, C-15,15′), 25.5 (2CH_3_, C-16,16′), 17.9 (2CH_3_, C-17,17′), 17.1 (2CH_3_, C-18,18′), 13.1 (2CH_3_, C-19,19′), 13.0 (2CH_3_, C-20,20′).

### 2.2. Molecular Docking Studies

**Figure 3:**
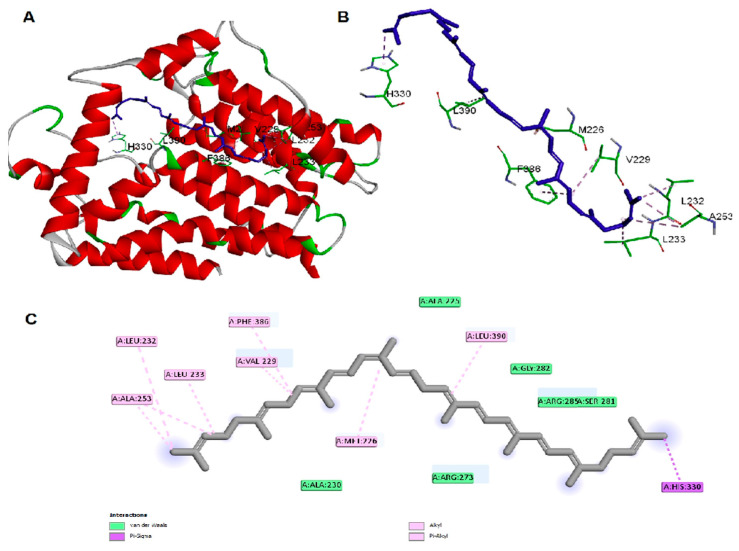
Docking of lycopene in *K. pneumoniae* lipid A core-O-antigen ligase (LAL) (**A**) whole protein view (**B**) active site view, and (**C**) the 2D schematic presentation of interaction forces.

**Figure 4:**
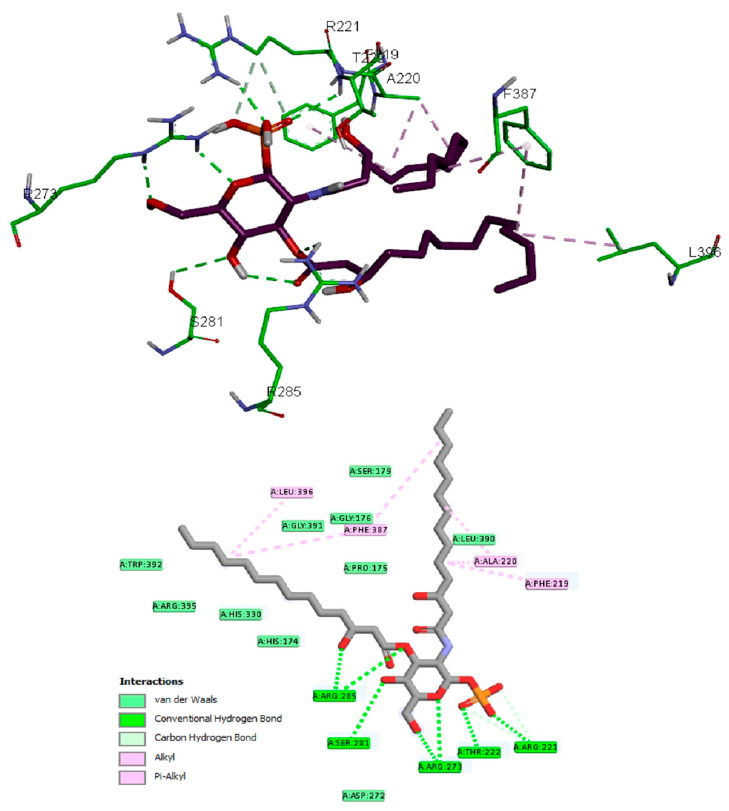
Docking of LAM in *K. pneumoniae* lipid A core-O-antigen ligase (LAL); active site view (**Top**) and the 2D schematic presentation of interaction forces (**Bottom**).

**Figure 5:**
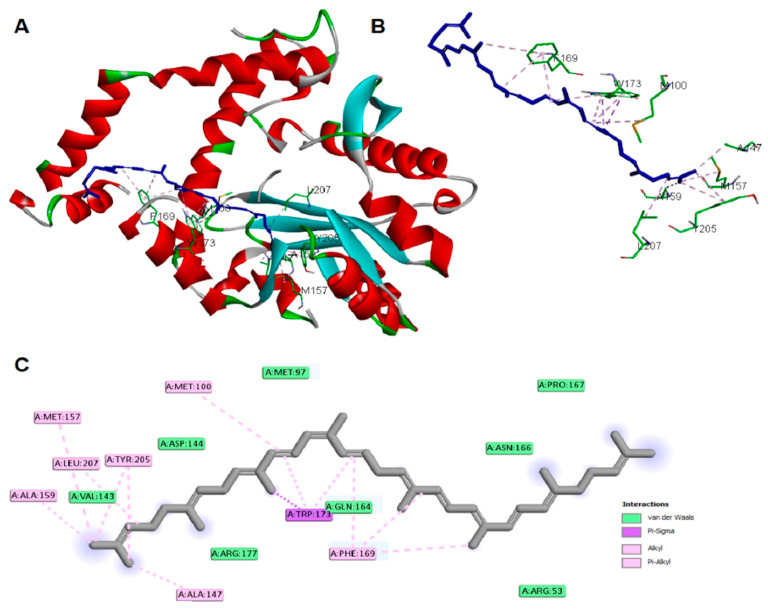
Docking of lycopene in *K. pneumoniae* lipid A biosynthesis myristoyl transferase (LBMT) (**A**) whole protein view (**B**) active site view, and (**C**) the 2D schematic presentation of interaction forces.

**Figure 6:**
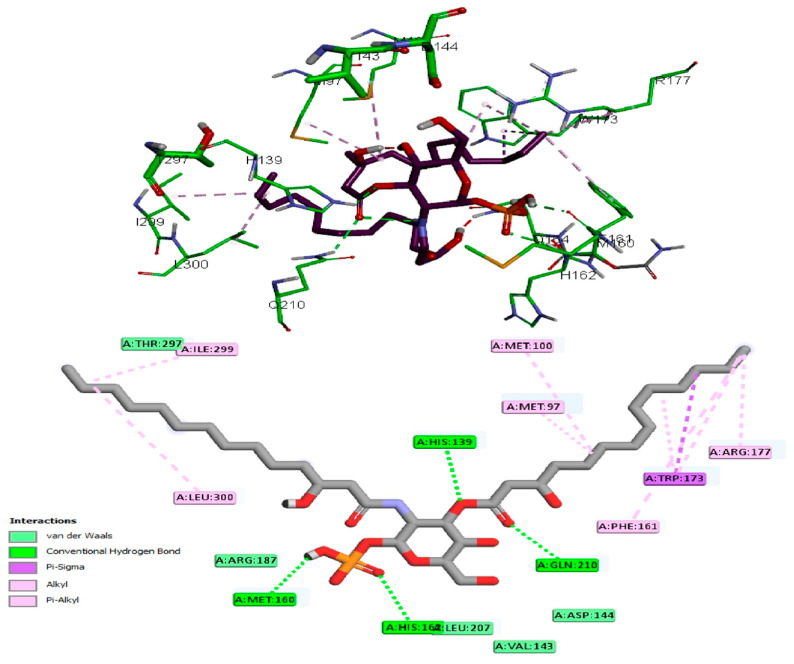
Docking of LAM in *K. pneumoniae* lipid A biosynthesis myristoyl transferase (LBMT); active site view (**Top**) and the 2D schematic presentation of interaction forces (**Bottom**).

The antibacterial activity of lycopene on *K. pneumoniae* was suggested to be based on its reported lipidemic lowering activity [20] and its activity on different lipases involved in lipoprotein metabolism [20,21]. Furthermore, there is a connection between sphingolipids/cholesterol and both expression and production of the *K. pneumoniae* capsule lipopolysaccharide [22]. In addition, the structural similarity between lycopene and sphingolipids lipid core monomers suggests the role of lycopene in interfering with one or more proteins involved in the production of capsule polysaccharides. Thus, a molecular docking approach was employed to study lycopene binding affinity and binding mechanisms of *K. pneumoniae* lipid A core-O-antigen ligase (LAL) and lipid A biosynthesis myristoyl transferase (LBMT). The ligands showed high and similar binding affinities to the target proteins, as indicated by the given docking scores (Table 1). The binding of lycopene and lipid A monomer (LAM) unit adapted similar site with either hydrophobic interactions, hydrogen bonding, or both. Docking of both ligands into the LAL receptor revealed high affinity near the eccentric of the protein (Figure 3). The hydrophobic interactions comprise the only forces engaging lycopene with LAL, while LAM interacted with H-bonds in addition to the main hydrophobic interactions (Figure 4). Similarly, docking of the two ligands into the active site of the LBMT enzyme revealed that hydrophobic interactions were the common interaction forces in both ligands (Figure 5 and Figure 6). Furthermore, four H-bonds were observed between the sugar part of LAM and the active site residues.

After all, both lycopene and LAM showed a good affinity for the studied proteins enrolled in the production of capsule lipopolysaccharide of *K. pneumoniae* suggesting the possible mechanisms for the antibacterial activity of lycopene.

### 2.3. Assay of Lycopene

The UV spectrophotometric assay method was successfully utilized to quantify lycopene in the analyzed samples. The constructed standard curve was linear in the 15–50 µg/mL concentration range with a correlation coefficient (R^2^) of 0.999. The method was validated, and the intraday recorded % recovery ranged from 97.1 to 102.2% of the nominal values. The computed relative standard deviation values ranged from 0.5 to 1%. The lower limit of detection and lower limit of quantification was calculated to be 1.1 µg/mL and 3.3 µg/mL, respectively.

### 2.4. Particle Size (PS) and Zeta Potential (ZP) Analysis

**Figure 7:**
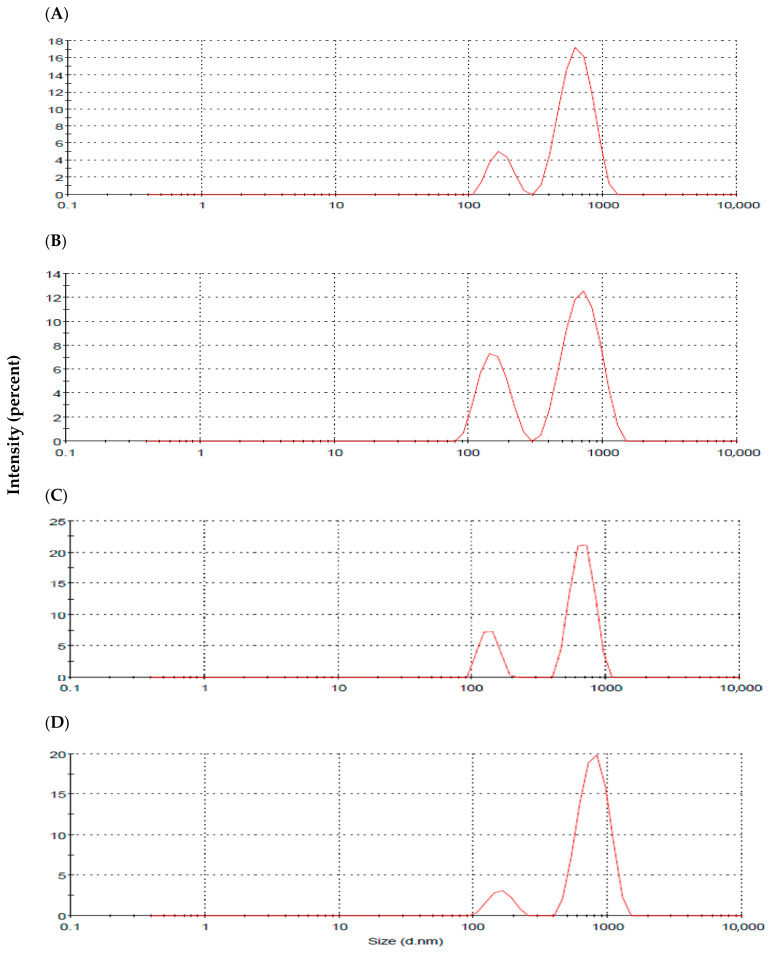
Particle size distribution graphs for F1 (**A**), F2 (**B**), F3 (**C**) and F4 (**D**) formulations.

The recorded vesicular size, polydispersity index (PDI), and ZP of the prepared formulations are presented in Table 2, with the particle size distribution shown in Figure 7. The vesicle size of the plain niosomes (F1) was 587 ± 74 nm, with a PDI value of 0.762. The recorded PDI value reflects the heterogenicity of the niosomal dispersion prepared by adopting the sonication method. This was further verified with the recorded bimodal size distribution graph (Figure 7). Initial incorporation of sodium cholate (NaCh) in bilosomes formulation (F2) resulted in the reduction of the vesicular size, with the PDI value being lowered compared to bile-salts-free niosomes (F1). The bimodal size distribution pattern was preserved with an improved intensity of the lower-sized particles (Table 2, Figure 7). Further increase in bile salt concentration in the constructed bilosomes (F3 and F4) resulted in vesicle enlargement with elevated PDI values. With regard to ZP, all the prepared vesicles were negatively charged with ZP values ranging from −37.3 ± 4.4 to −43.9 ± 5.3 mV, indicating their stability (Table 2).

### 2.5. Entrapment Efficiency Determination

Estimating lycopene entrapment efficiency in the prepared systems involved determining the free unentrapped drug following centrifugation. The estimated %EE values were 94.3%, 93.2%, 89.1%, and 87.6% for F1, F2, F3, and F4 formulations, respectively. The results highlighted the effect of increasing NaCh content to lower lycopene entrapment in the prepared vesicular dispersions. This effect was non-significant between F1 and F2 formulations, with the calculated %EE significantly reduced for F3 and F4 formulations (*p* < 0.01).

### 2.6. In Vitro Drug Release Study

**Figure 8:**
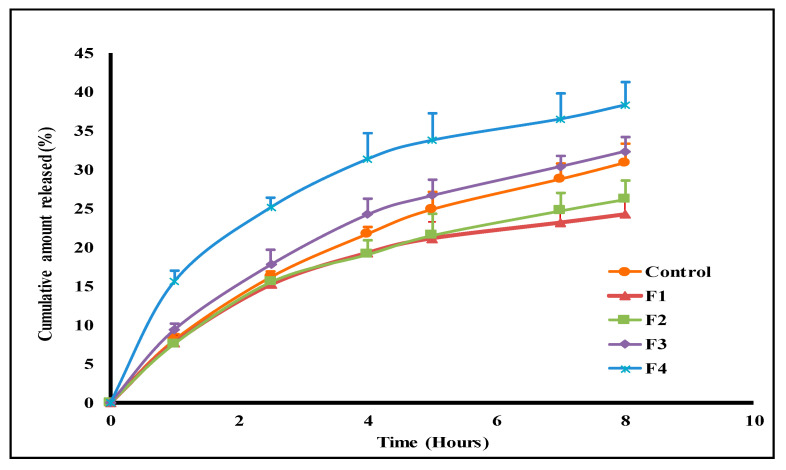
In vitro release profiles of lycopene from the tested formulations and aqueous lycopene suspension (control).

The release profiles of lycopene from the aqueous control suspension and the tested vesicular systems are illustrated in Figure 8. These profiles were utilized to compute the drug release efficiency values shown in Table 2. The release efficiency (%RE) for the unprocessed lycopene was 19.7%, with the cumulative % amount released reaching 30.9 ± 2.4% after 8 h (Table 2, Figure 8). Incorporating lycopene into niosomes (F1) further extended lycopene release behavior relative to the drug control. The recorded cumulative amount released (%) after 8 h was reduced to 24.3 ± 2%, with the calculated %RE being decreased to 16.9%. Bilosomal lycopene encapsulation fastened drug release relative to the bile-salts-free formulation (F1), with the computed %RE values being 17.4%, 21.3%, and 27.6% for F2, F3, and F4 formulations, respectively (Table 2). The computed similarity factor (F2) values revealed release profiles similarity between F1, F2, and F3 formulations (F2 ˃ 50), with the F4 formulation being dissimilar to both F1 and F2 formulations (F2 < 50). The release kinetics of lycopene from the tested systems were estimated through linear regression analysis after data fitting to different kinetic models. The calculated correlation coefficient (R^2^) values are presented in Table 3. The fitting reflected Higuchi-based liberation of lycopene from all tested formulations.

### 2.7. Transmission Electron Microscopy (TEM)

**Figure 9:**
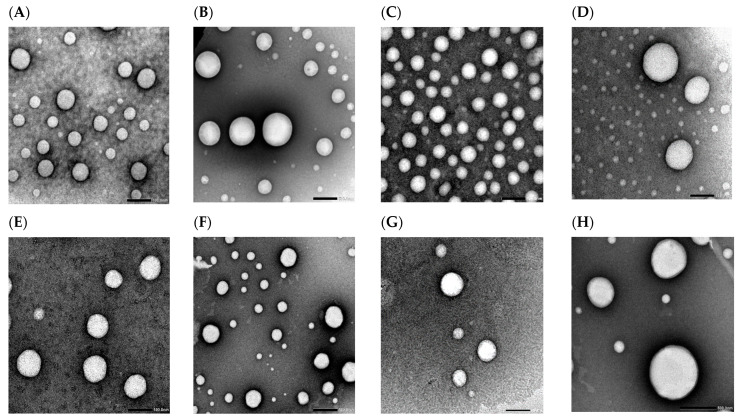
Transmission electron micrographs for F1 (**A**,**B**), F2 (**C**,**D**), F3 (**E**,**F**) and F4 (**G**,**H**) formulations.

Representative transmission electron micrographs of niosomes (F1) and constructed bilosomes formulations (F2, F3, and F4) are shown in Figure 9. These exposed spherical nanostructured vesicles with no agglomeration. The representative captures for bile-salts-free F1 formulation revealed heterogenicity of vesicular size distribution with two different size populations being recorded (Figure 9A,B). The same pattern was recorded for the optimized bilosomes formulation (F2). However, there was a remarkably increased intensity for the smaller vesicles’ population and fairly improved homogeneity relative to F1 niosomes (Figure 9C,D). The captured micrographs for F3 and F4 formulations revealed no morphological change in the vesicular structure upon increasing bile salts ratio (Figure 9E–H). Size enlargement was demonstrated at higher bile salts concentration which confirmed the recorded DLS data (Figure 9H).

### 2.8. In Vitro Antibacterial Activity

**Figure 10:**
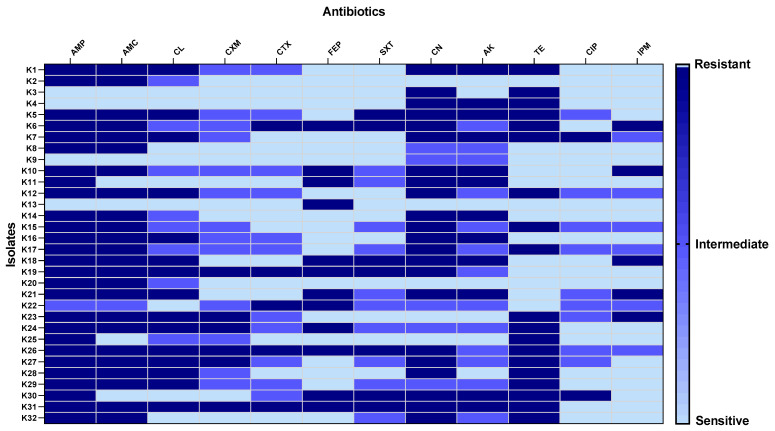
Heat map showing antibiotic susceptibility of *K. pneumoniae* isolates. AMP: ampicillin, AMC: amoxicillin/clavulanic acid, CL: cephalexin, CXM: cefuroxime, CTX: cefotaxime, FEP: cefepime, SXT: cotrimoxazole, CN: gentamicin, AK: amikacin, TE: tetracycline, CIP: ciprofloxacin, and IPM: Imipenem.

*K. pneumoniae* isolates exhibited antibiotic resistance to various antibiotics, as shown in Figure 10.

Regarding the susceptibility of *K. pneumoniae* isolates to free lycopene and its formulations, the tested isolates exhibited susceptibility to free lycopene. Moreover, the medicated formulations (F1, F2, F3, F4) revealed antibacterial susceptibility compared to their corresponding non-medicated formulations, which did not show any antibacterial activity, as shown in Table S1. A representative example of the inhibition zone of free lycopene, F2 formulation, and non-medicated F2 formulation is supplied in Figure S1.

The minimum inhibitory concentrations (MIC) values were determined for the free lycopene and its free formulations, presented in Table 4. Both F1 and F2 exhibited the lowest MIC range of 8 to 32 µg/mL compared to the free lycopene and the other two formulations (F3 and F4).

#### Scanning Electron Microscopy (SEM)

**Figure 11:**
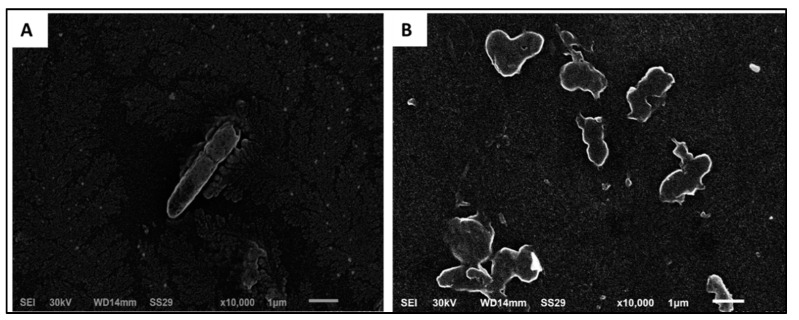
Scanning electron micrographs of representative *K. pneumoniae* isolates (**A**) before and (**B**) after treatment with F2 bilosomes.

SEM analysis of the effect of F2 formulation on the tested isolates was carried out, and a representative example is shown in Figure 11. As shown in Figure 11, the F2 formulation resulted in the deformation and lysis of the tested isolates.

### 2.9. In Vivo Antimicrobial Activity

#### 2.9.1. Survival Rate and Count of Colony-Forming Unit (CFU/mL)

**Figure 12:**
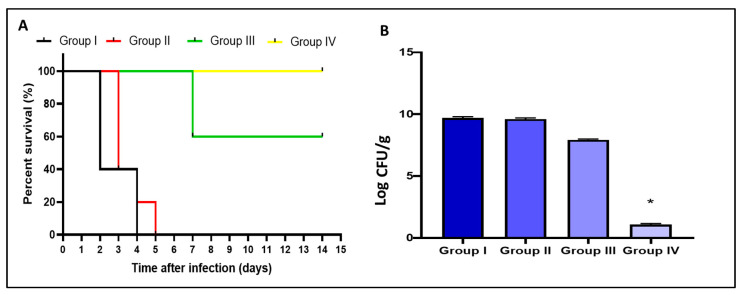
Survival curve (**A**) and the number of CFU/g lungs (**B**) of the different groups. The symbol (*) represents a significant difference (*p* < 0.05) in comparison with the experimental groups I, II, and III.

We constructed the Kaplan–Meier survival curve as shown in Figure 12A. Three rats in group I died after two days, and the other two rats died after four days. Regarding group II, three rats died after three days, one rat died after four days, and one rat died after five days. Only two rats died after one week in group III, and all the rats remained alive till the 14th day in group IV. Moreover, the number of CFU/g lung tissues was counted in all tested groups. F2 formula significantly reduced (*p* < 0.05) the number of CFU/g lungs, as shown in Figure 12B.

#### 2.9.2. Haematoxylin and Eosin (H&E) and Masson’s Trichrome Staining

**Figure 13:**
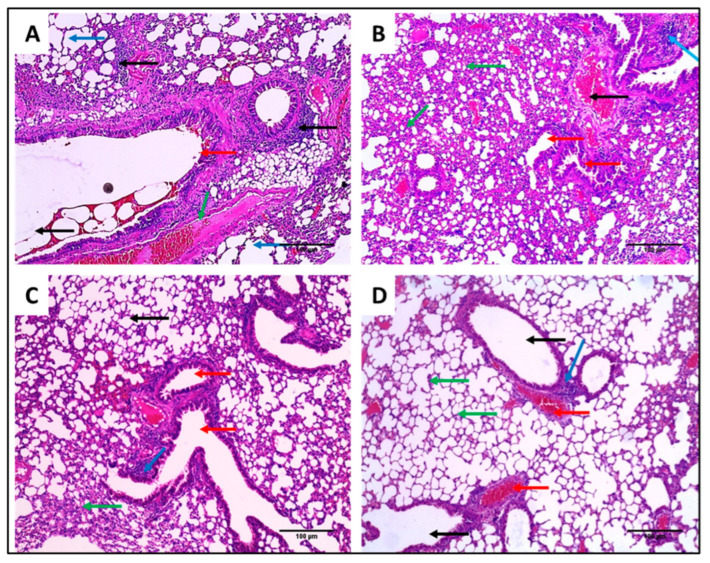
H&E staining of lung sections of (**A**) Group I showing dilated destructed alveoli (emphysema) (blue arrows) surrounded by fibrosis and dilated bronchioles (red arrow) surrounded by chronic inflammation (black arrow) and congested vessel (green arrow) (×200). (**B**) Group II showed dilated destructed bronchioles (red arrows) surrounded by marked alveolar fibrosis (green arrows), chronic inflammation (blue arrow), and congested vessels (black arrow) (×200). (**C**) Group III showed focal inflammation (blue arrow) with average-sized bronchioles (red arrows) surrounded by normal-sized alveoli (black arrow) with residual areas of alveolar fibrosis (green arrow) (×200). (**D**) Group IV showed minimal inflammation (blue arrow) surrounded by normal-sized bronchioles (black arrow) and normal-sized alveoli (green arrows) with very few congested vessels (red arrows) (×200).

**Figure 14:**
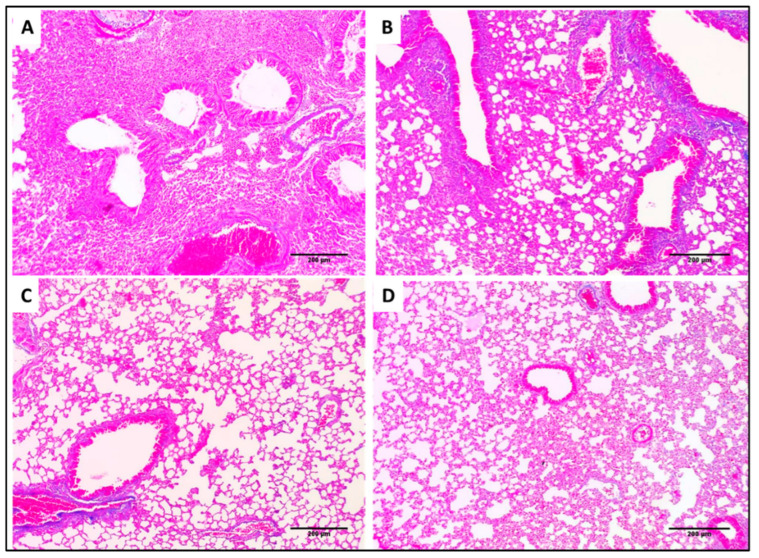
Lung sections stained with Masson’s stain of (**A**) Group I showed marked fibrosis (×100). (**B**) Group II showed marked fibrosis (×100). (**C**) Group III showed minimal fibrosis (×100). (**D**) Group IV showed normal lung tissues without fibrosis (×100).

Lung tissues of the different experimental groups stained with H&E and Masson’s trichrome stain are shown in Figure 13 and Figure 14, respectively.

## 3. Discussion

Recently, *K. pneumoniae* has progressively developed resistance to most commonly used antibiotics, especially in hospitals [23]. Herein, 32 *K. pneumoniae* isolates were obtained from different specimens from patients admitted to Tanta University Hospitals. We performed antibiotic susceptibility testing for these isolates and found that 65.63% of the isolates were MDR. Even though this relatively elevated incidence of MDR *K. pneumoniae* isolates was reported by previous studies [23,24,25,26], our study is the first to report the high prevalence of MDR *K. pneumoniae* isolates in Tanta city, Egypt. Owing to the difficulty of treating MDR bacteria, it is essential to find new therapeutic approaches to decrease its morbidity and mortality rates.

In the current study, we investigated the antimicrobial activity of lycopene, and we aimed to prepare a suitable formulation to enhance its in vivo bioavailability and antibacterial activity against *K. pneumoniae* clinical isolates after oral administration. Furthermore, the molecular docking approach was employed for studying lycopene binding affinity and binding mechanisms on two proteins involved in capsule lipopolysaccharide production in *K. pneumoniae*. Docking results demonstrated *K. pneumoniae* lipid A core-O-antigen ligase (LAL) and lipid A biosynthesis myristoyl transferase (LBMT) as potential molecular targets explaining the antibacterial activity of lycopene against *K. pneumoniae*.

The developed UV spectrophotometric analysis method was sensitive, selective, and precise enough to quantify the amounts of lycopene. Bilosomes formulations were fabricated for oral lycopene delivery considering the substantial advantages of bilosomal encapsulation for improvement of oral drug delivery. These include improved colloidal gastrointestinal stability and permeation enhancement effect of incorporated bile salts [12,13]. The particle size of bilosomes exerts a considerable effect on both their in vitro and in vivo performance [11]. PDI was adopted as an indicator for homogeneity of the prepared vesicular dispersions. The recorded PDI values for all the prepared formulations were greater than 0.5, reflecting the heterogenicity of the prepared systems.

Additionally, particle size distribution graphs revealed a bimodal distribution pattern (Figure 7). This was formerly expected for the vesicles prepared by bath sonication following hydration of pre-vesicular concentrate. Other investigators previously reported similar behavior for vesicles prepared using the same technique [27,28]. Bile-salts-free niosomal vesicles (F1) revealed bimodal size distribution, with the average particle size being 587 ± 74 nm (Figure 7A). The literature highlighted a similar colloidal size range for niosomes prepared using non-ionic surfactants [29].

The incorporation of bile salts in the prepared vesicles exhibited a crucial impact on the recorded Z-average, PDI, and ZP. The relative effect depended mainly on the bile salts concentration. Initial incorporation of NaCh in F2 formulation increased the intensity of the lower-sized vesicles with an overall reduction of the computed Z-average compared to plain niosomes (Figure 7B). This can be explained by the reduced interfacial tension and improved vesicular flexibility upon the incorporation of bile salts [30,31]. The recorded PDI value revealed improved homogeneity, with the dispersion being free from any aggregates. Further increase in NaCh concentration in the prepared bilosomes resulted in the formation of enlarged vesicles. This can be attributed to the increased bilayers repulsion at higher content of negatively charged bile salts [32]. Moreover, the bulky steroidal structure of NaCh may play a role in the increased vesicular size at higher concentrations [33,34]. A similar pattern of the effect of bile salts content on the recorded vesicular size and PDI of bilosomes was reported by other investigators [12]. The higher computed PDI values for F3 and F4 formulations were taken as an indicator for the presence of larger micron-sized particles and/or aggregates out of the instrumentally graphed size distribution scale.

The computed ZP values reflected the stability of all the prepared formulations. Generally, systems with ZP around ±30 mV are considered to possess enough charge to prevent vesicular aggregation, triggering system stability [29,32]. Increasing NaCh concentration in bilosomes augmented the recorded ZP value, with the increasing extent being significant in F2 and F3 formulations compared to plain niosomes (*p* > 0.01). This was expected regarding the ionic nature of incorporated NaCh [34].

Regarding lycopene entrapment in the prepared formulations, the calculated results revealed high %EE values, ranging from 87.6% to 94.3%. When taking into consideration the highly hydrophobic lycopene structure, the recorded high encapsulation efficiency can be explained. The impact of physicochemical properties of drugs on their entrapment efficiency into vesicular carriers was reported in the literature [35]. It is essential to highlight the reduced drug entrapment with increasing NaCh concentrations. This was noticed as a trend at the initial addition of NaCh in F2 bilosomes relative to plain F1 niosomes.

Further increase in NaCh concentration (F3 and F4) resulted in a remarkable decrease in %EE despite being larger in size compared to F1 and F2 formulations. This was attributed to partitioning the incorporated bile salts into the dispersion medium with subsequent drug micellization and improved solubility in the aqueous compartment of the vesicles reducing %EE. The effect of bile salts concentration on the %EE in bilosomes was similarly reported by other investigators [32]. Additionally, fluidization of the vesicular lipid bilayer at higher bile salt content increases drug release and lowers entrapment efficiency [11].

This effect was further confirmed with the in vitro lycopene release study from the prepared vesicular systems. This was accomplished employing 40% ethanolic solution incorporating 0.3% *w*/*v* tween 80. This was adopted to maintain sink conditions throughout the release study [36]. The recorded release profile of the free lycopene aqueous dispersion revealed slow drug release, with only 30.9% of the drug being liberated over 8 h (Figure 8). This pattern was attributed to the highly hydrophobic lycopene structure. Niosomal encapsulation resulted in further sustained drug release from F1 formulation compared to free lycopene due to preferential drug partitioning into the vesicular lipid bilayer. Similar behavior of sustained lycopene release after incorporation into lipid nanostructures was previously reported in the literature [37,38]. The initial addition of NaCh to the prepared vesicular system (F2 bilosomes) resulted in a non-significant alteration of the drug release parameters relative to the bile-salts-free niosomes, with the recorded release profiles of F1 and F2 being similar (F2 value = 92%).

Further, increased bile salts concentration in F3 and F4 formulations augmented lycopene release relative to F1, F2, and free lycopene aqueous dispersion (Figure 8). The computed similarity factor values for F4 bilosomes reflected dissimilarity to both F1 and F2 formulations. These recorded results coincide with the calculated %EE of lycopene in the prepared systems (Table 2). This was explained based on improved drug solubility in the dispersion medium and vesicular lipid bilayer fluidization with increased NaCh content in the prepared system [11].

The release kinetics of lycopene from all the prepared formulations followed the Higuchi model suggesting matrix diffusion-based drug release. These findings are consistent with the release kinetics recorded by other investigators for niosomes encapsulated drugs [39,40].

Morphological characterization of the constructed formulations was performed utilizing TEM analysis. The captured transmission electron micrographs revealed no difference between the fabricated formulations regarding the vesicle morphology. Bile-salts-free niosomes and bilosomes formulations were physically similar, showing spherical vesicles free from aggregation. A bimodal size distribution pattern was noticed for the micrographed formulations, which coincide with the formerly recorded PS and PDI results. Improved system homogeneity and smaller vesicular size were noticeable for F2 bilosomes micrographs (Figure 9C,D) relative to the bile-salts-free niosomes (Figure 9A,B). Moreover, vesicular size enlargement was demonstrated at higher bile salts concentration (Figure 9H). This would further confirm the recorded DLS results for these formulations.

MIC range values were determined for unprocessed lycopene and the prepared formulations. Both F1 and F2 had the lowest MIC range of 8 to 32 µg/mL compared to free unprocessed lycopene and the other two formulations, F3 and F4. A lower MIC value denotes that less drug concentration is needed to inhibit bacterial growth. Thus, the lower MIC values are taken as an indicator of more efficient antimicrobial activity [41]. Thus, both F1 and F2 modulated the antibacterial activity of lycopene against the tested *K. pneumoniae* isolates. Furthermore, SEM revealed deformation and lysis of the F2 bilosomes-treated isolates. Disruption of the cell morphology with cell degradation and cell membrane damage is a mechanism of action of many antimicrobials [42]. Based on these findings, together with the recorded PS, PDI, ZP, %EE, and %RE, we investigated the in vivo antibacterial activity of optimized F2 formulation in infected mice.

Interestingly, the F2 formula reduced the inflammation and congestion in the treated mice’s lung tissues, resulting in normal-sized bronchioles and alveoli with very few congested vessels. In addition, it resulted in a significant reduction in pulmonary fibrosis. Pulmonary fibrosis is a pathological condition in the lung which occurs when the lung tissues are damaged and scarred due to different causes such as bacterial infections. These stiff and thickened tissues affect lung function.

Overall, the recorded data in this study highlighted the in vitro and in vivo antimicrobial potential of bilosomes-encapsulated lycopene against *K. pneumoniae* isolates. These bile-salts-modified vesicular systems have been reported to exert a membrane fluidizing effect providing greater flexibility to biological membranes, including bacterial cell walls [43]. This effect was further confirmed regarding the recorded SEM micrographs, which revealed the presence of ruptured cells after bilosomes treatment. The in vivo performance of bilosomes can be attributed to the enhancement of intestinal membrane permeability [12]. This may be attributed to the potential of incorporated bile salts to fluidize membrane lipid bilayer with subsequent improvement of intestinal permeation [44,45]. Moreover, the possibility of intact nano-vesicles translymphatic absorption after oral administration should be considered. This has been reported to be the most probable factor for enhanced oral drug bioavailability from vesicular carriers [46]. However, this requires further investigations.

## 4. Materials and Methods

### 4.1. General Chemicals and Materials

Cholesterol and Span 60 were purchased from Oxford Lab Fine Chem LLB, India. In addition, NaCh was procured from Loba Chemie, India. Ethanol and Tween 80 were obtained from El-Nasr Pharmaceutical Chemicals Company, Cairo, Egypt. Cellulose dialysis tubing and the other chemicals utilized in this study were attained from Merck, USA. The utilized antibiotic disks and media were purchased from Oxoid, UK. For column chromatography (CC), we employed Silica gel F254 (Merck, 70–230 mesh)

Digital FT-Avance III NMR spectrometer (Bruker, Karlsruhe, Germany) recorded NMR spectra at 400 MHz for ^1^H and 100 MHz for ^13^C and DEPT. CDCl_3_ was utilized to dissolve the NMR sample. The chemical shifts were normalized using solvent resonances. Thermo Scientific’s ISQ Quantum Access MAX Triple Quadrupole system, Xcalibur 2.1 software, and USA Mass Spectrometer were utilized for the ESI-MS.

### 4.2. Isolation of Lycopene

Tomato or *Lycopersicon esculentum* L. (Family Solanaceae) was selected from a local farm on 5 February 2021. The plant identity was confirmed by Dr. Esraa Ammar, Plant Ecology, Tanta University. Tomato powder was made by crushing tomatoes (one kg) in a mixer and then lyophilized. The lyophilized powder (200 g) was extracted by n-hexane: methylene chloride (1:1) then the extract was concentrated using a rotary evaporator. The obtained residue was then column chromatographed isocratically with methylene chloride to isolate lycopene.

### 4.3. Molecular Docking Studies

*K. pneumoniae* lipid A core-O-antigen ligase LAL (Uniprot code: A0A193SFX0) [47] was modeled by Swiss-model server [48] using the WaaL O-antigen ligase (PDB Code: 7TPG) [49] as a template. *K. pneumoniae* lipid A biosynthesis myristoyl transferase LBMT (Uniprot code: A0A2D1HIK6) was modeled by Swiss-model server using the Lipid A secondary acyltransferase LpxM from *Acinetobacter baumannii* (PDB Code: 5KN7) [50] as a template. The docking study on lycopene and lipid A monomer (LAM) was carried out using AutoDock Vina [51]. Ligand structures were drawn into Marvin Sketch V22.2 [52], and the most energetically favored conformer was exported as (*.pdb) file format. The procedure for docking simulation was according to our previous study steps [53]. The center and size of the grid box to define the active site for each receptor were (X, Y, Z: 129.3, 150.5, 133.6) and (X, Y, Z: 15, 24, 23), respectively. The 3D visualization and 2D schematic presentation were generated by the Discovery Studio client [53].

### 4.4. Assay of Lycopene

Lycopene analysis utilized a UV spectrophotometer (Thermo Fisher Scientific, Waltham, MA, USA) with quantification directed at 446 nm. Stock ethanolic lycopene solution (500 mg/mL) was prepared and subsequently diluted with ethanol into concentrations ranging from 15 to 50 µg/mL. The standard calibration curve was constructed by plotting the recorded absorbance values versus drug concentration. The computed linear curve equation was then utilized for routine drug quantification. The method was validated according to the International Conference on Harmonization (ICH) guidelines (ICH, 1996). The validation parameters covered linearity, accuracy, precision, the lower limit of detection (LOD), and the lower limit of quantification (LOQ).

### 4.5. Preparation of Bilosomes

Table 5 presents the composition of the tested niosomes and bilosomes formulations. The prepared plain niosomal dispersion (F1) comprised Span 60 and cholesterol at a weight ratio of 1:0.25 *w*/*w*. NaCh was incorporated in the constructed bilosomal dispersions at molar concentrations of 0.01, 0.025, and 0.04 M for F2, F3, and F4, respectively. The technique adopted for preparation was previously clarified in literature [54]. Briefly, Span 60, cholesterol, and NaCh (if present) were dispersed in 2.5 g of ethanol before warming on a water bath adjusted at 65 ± 1 °C. The dispersion was kept at this temperature till clarity. Lycopene was added to the formulations before the addition of 2.5 mL of warm water with gentle shaking to produce homogenous dispersion. Cooling down of the resulting mixtures was allowed, with the mixing continued at ambient temperature to produce proconcentrate gel. The prepared formulations were subsequently diluted with 10% of the set water amount to yield consistent niosomal dispersion. The prepared niosomes were left overnight before complete hydration to the required volume and for 40 min using a bath sonicator (Elmasonic S23, Germany).

### 4.6. Particle Size (PS) and Zeta Potential (ZP) Analysis

Bilosomal dispersions were appropriately diluted with deionized water before being sonicated for a few minutes to generate homogenously vesicular dispersion devoid of aggregates. The mean PS, polydispersity index (PDI), and ZP of the constructed systems were analyzed employing the dynamic light scattering (DLS) technique with the aid of Zetasizer Nano-ZS (Malvern Instruments Ltd., Malvern, UK). All the measurements were conducted in triplicates (*n* = 3) at 25 °C. The PS data are presented as Z-average ± standard deviation (SD), with the PDI values computed using the instrumental software.

### 4.7. Entrapment Efficiency Determination

The %EE of lycopene in the constructed vesicular systems was calculated indirectly through the measurement of the un-entrapped free lycopene in the dispersion medium. Three milliliters of the prepared vesicular dispersions were exposed to ultra-centrifugation using a cooling centrifuge (3–30 K, Sigma laborzentrifugen, Osterode am Harz, Germany) at 20,000 rpm for 90 min at 4 °C [55,56]. The obtained supernatant was carefully decanted and diluted with ethanol (if required) before lycopene quantification using the validated UV-VIS spectrophotometric analytical method at λmax 446 nm. The %EE was mathematically computed applying the following equation:

$$Entrapment\;efficiency\left(\%\right)=\left[\frac{Ct-Cf}{Ct}\right]\times 100$$

where *Ct* is the total lycopene amount added to 3 mL vesicular dispersion (1.8 mg) and *Cf* is the amount of un-entrapped free lycopene.

### 4.8. In Vitro Drug Release Study

In vitro lycopene release from the constructed bilosomes, bile salt-free niosomal dispersion, and aqueous lycopene dispersion (control) was investigated using the dialysis method [54]. The immediately hydrated and sonicated vesicular dispersions were dialyzed against 40% ethanolic solution. In order to maintain sink conditions during the study, tween 80 (0.3% *w*/*v*) was added to the release medium [36,37]. Concisely, dialysis bags (cellulose tubing, cutoff 14,000 Da) were soaked overnight in distilled water to confirm complete membrane swelling and constant pore size throughout the release experiment. Dialysis bags were then properly cut and filled with 3 mL of the tested systems. The properly tied filled sacs were immersed in the release medium (50 mL) and maintained at 37 ± 0.5 °C with a constant mild stirring intermittently applied. Samples were withdrawn from the release medium at predetermined time points for 8 h and replenished with fresh medium preheated to 37 °C of equivalent volume. The drug content in each collected sample was quantified using the formerly mentioned UV-VIS spectrophotometric method. The release studies were conducted in triplicates, and the cumulative lycopene amount released expressed as a percentage were plotted as a function of time to construct release profiles. These profiles were employed for the calculation of the release efficiency (RE), employing the non-linear trapezoidal rule from the computed area under the release plot at a time (*t*) relative to the overall area assuming 100% drug release at the same time [57]. Furthermore, the release kinetics of lycopene from the fabricated vesicular systems was estimated through data fitting to zero and first Higuchi and Korsmeyer–Peppas models [58,59]. Linear regression analysis was followed for the computation of the correlation coefficient (R^2^) value for each model. The similarity factor values (F2) were calculated to compare the overall lycopene release profiles, with (F2) values in the range of 50–100% being taken as an indicator of the two profiles’ similarity. This was computed using the following equation:

$$F2=50\times \log\{{\left[1+\frac{1}{n}\overset{n}{\underset{t=1}{\sum }}\left(R_{t}-T_{t}\right)\right]}^{-0.5}\}\times 100$$

where (R_t_) and (T_t_) represent amounts of lycopene released at time *t* and (*n*) represents the number of data points.

### 4.9. Transmission Electron Microscopy

Morphological characterization of the fabricated formulations was performed utilizing TEM analysis. This was conducted using TEM (JEOL, JSM1400 PLUS, Tokyo, Japan). The tested vesicular dispersions were properly diluted with double filtered water. One drop of each sample was loaded on a copper grid and stained with saturated uranyl acetate solution in 70% ethanol. The stained samples were then dried at 25 °C for 10 min preceding TEM observation and capturing photomicrographs of the scanned systems.

### 4.10. In Vitro Antimicrobial Activity

#### 4.10.1. Bacteria

Thirty-two *K. pneumoniae* isolates were obtained from the laboratories of Tanta University Hospitals. They were inspected microscopically and identified using biochemical tests. *Klebsiella pneumoniae* (ATCC 13883) was utilized as a reference strain.

#### 4.10.2. Antibiotics Susceptibility Testing

This test was carried out using the Kirby-Bauer method [60,61], and the diameters of the inhibition zones around the antibiotic disks were measured in millimeters (mm). In brief, the antibiotic disks were placed onto the plates after spreading the bacterial suspensions (0.5 McFarland) on the surfaces of Muller-Hinton agar plates. Then, the plates were incubated at 37 °C for 24 h. The utilized antibiotic discs were ampicillin (AMP, 10 μg), amoxicillin/clavulanic acid (AMC, 20/10 μg), cephalexin (CL, 30 μg), cefuroxime (CXM, 30 μg), cefotaxime (CTX, 30 μg), cefepime (FEP, 30 μg), cotrimoxazole (SXT, 1.25/23.75 μg), gentamicin (CN, 10 μg), amikacin (AK, 30 μg), tetracycline (TE, 30 μg), ciprofloxacin (CIP, 30 μg), and Imipenem (IPM, 10 μg). *K. pneumoniae* isolates were regarded to be multidrug-resistant (MDR) when they were non-susceptible to at least one drug in three or more antibiotic classes [62].

#### 4.10.3. Antibacterial Susceptibility Testing of the Free Lycopene and Its Formulations

Free lycopene, its formulations (F1, F2, F3, F4), and their corresponding non-medicated formulations were screened for their antimicrobial activity against the tested isolates using the disc agar diffusion method as previously described. Dimethyl sulfoxide (10%) and ciprofloxacin were utilized as negative and positive controls, respectively.

#### 4.10.4. Determination of MICs

MIC values of the free lycopene and its formulations were determined using the resazurin-based broth microdilution method in 96 well microtitration plates as previously described [63,64].

#### 4.10.5. Scanning Electron Microscopy

The alternations in the morphology of the treated isolates after treatment with F2 bilosomes formulation were investigated compared to the non-treated cells using SEM (Hitachi, Chiyoda, Japan).

### 4.11. In Vivo Antimicrobial Activity

#### 4.11.1. Animals

Forty male Swiss albino mice, with weights of 18–24 g, were used for the in vivo experiment in our study. We followed the ethical guidelines of the Faculty of Pharmacy Research Ethical Committee, Tanta University (TP/RE/04-22-PH-004). The animals had free access to food and water, and they were kept under a 12 h light-dark period at room temperature.

#### 4.11.2. Experimental Model

The antibacterial efficacy of lycopene and its bilosomal dispersion (F2) was investigated in vivo using a lung infection model [65,66]. In brief, the bacterial suspension, with a concentration of 10^7^ CFU/mL, was utilized to induce lung infection in mice. Each mouse was lightly anesthetized with isofluorane before infection and received 25 µL of the bacterial suspension in both nares. Mice were then grouped equally into four groups: group I (control), which did not receive any treatment. Group II received the placebo or non-medicated formulation, group III received free lycopene, and group IV received lycopene formulation (F2). The dilution of the prepared bilosomes and orally administered volume to each animal were adjusted to deliver a lycopene dose equivalent to 13.2 mg/kg, estimated based on the FDA dose conversion tables [67]. All treatments were administered orally after 4 h, 24 h, and 48 h. On the third day of the experiment, five mice were anesthetized from each group and euthanized. Lung tissues were obtained for histological examination, collagen studies, and determination of the bacterial burden (number of CFU/g). The remaining rats were left for 14 days to determine the survival rate.

#### 4.11.3. Histological Assessment and Masson’s Stain

Lung tissues were fixed in formalin (10%) for 72 h [68], dehydrated, placed in paraffin wax, cut into thin sections (3 μm), and stained using H&E and Masson’s trichrome stains. Photos were captured by a digital camera.

### 4.12. Statistical Analysis

Data were revealed as mean ± SD. One-way analysis of variance (ANOVA), followed by a post hoc test (Tukey), was utilized to assess the differences among the experimental groups. Moreover, the Kaplan–Meier survival curve was utilized to estimate the survival of mice. The difference is significant if *p* < 0.05 using Prism version 8 (GraphPad Software, Inc, San Diego, CA, USA).

## 5. Conclusions

After isolating lycopene and performing molecular docking studies to assure its efficacy against *K. pneumoniae*, it was efficiently encapsulated into nanostructured bilosomes. The release pattern of the drug from the prepared bilosomal dispersions followed the Higuchi kinetics model. The physical stability of the constructed nano-vesicular system was indicated from the recorded zeta potential value with the captured TEM micrographs revealing spherical vesicles free from aggregates. Moreover, lycopene bilosomes exhibited an efficient in vitro antibacterial activity against multidrug-resistant *K. pneumoniae* isolates. Furthermore, the in vivo antibacterial activity of lycopene bilosomes was evaluated using a lung infection model in mice. Interestingly, it reduced the inflammation and congestion in the treated mice’s lung tissues, significantly reducing pulmonary fibrosis. The study thus introduced bilosomes nanovesicles with a high potential for optimizing oral delivery and in vivo efficacy of highly hydrophobic naturally isolated ingredients.

## Figures and Tables

**Table 1:** The grid box parameter (center and size) and docking binding affinity (Kcal/mL) of lycopene and lipid A monomer (LAM) for some proteins involved in producing *K. pneumoniae* lipopolysaccharide.

Receptor	Grid Box (x, y, z)		Affinity (kcal/mol)	
	Center	Size	Lycopene	LAM
LAL	129.3, 150.5, 133.6	15, 24, 23	−5.8	−5.4
LBMT	−14.8, −11.0, 10.0	19.5, 25.9, 22.0	−5.4	−5.6

**Table 2:** The recorded characteristics of the tested formulations.

Formulation	Z-Average (nm)	PDI *	ZP * (mV)	EE * (%)	RE * (%)
F1	587 (74.3)	0.762 (0.02)	−37.3 (4.4)	94.3 (0.5)	16.9 (1.5)
F2	485.8 (35.3)	0.522 (0.05)	−38.3 (4.0)	93.2 (0.6)	17.4 (1.4)
F3	653.1 (167.4)	0.747 (0.22)	−42.0 (5.3)	89.1 (0.12)	21.3 (1.9)
F4	672.7 (135.7)	0.756 (0.09)	−43.9 (5.3)	87.6 (1.2)	27.6 (2.3)
Control	**-**	**-**	**-**	**-**	19.7 (1.3)

* Poly dispersity index (PDI); zeta potential (ZP); % entrapment efficiency (EE) and % release efficiency (RE). Values between brackets are standard deviation (SD, *n* = 3).

**Table 3:** Correlation coefficient (R^2^) values for lycopene release profiles fitted to different release models.

Formulation	Zero-Order	1st Order	Higuchi	Korsmeyer–Peppas
F1	0.891 (0.046)	0.817 (0.085)	0.984 (0.008)	0.965 (0.012)
F2	0.902 (0.024)	0.848 (0.074)	0.986 (0.003)	0.965 (0.02)
F3	0.916 (0.02)	0.832 (0.101)	0.992 (0.002)	0.986 (0.007)
F4	0.835 (0.007)	0.838 (0.066)	0.981 (0.005)	0.979 (0.014)
Control	0.937 (0.007)	0.872 (0.04)	0.991 (0.002)	0.989 (0.004)

Values between brackets are SD (*n* = 3).

**Table 4:** MIC values of free lycopene and its formulations.

Isolate Code	MIC Value (µg/mL)					Isolate Code	MIC Value (µg/mL)				
	Free Lycopene	F1	F2	F3	F4		Free Lycopene	F1	F2	F3	F4
K1	1024	8	16	512	1024	K17	512	8	8	512	512
K2	1024	8	8	1024	512	K18	2048	16	16	1024	1024
K3	2048	32	16	1024	1024	K19	1024	16	8	512	1024
K4	512	32	16	512	512	K20	512	32	32	512	512
K5	2048	8	8	1024	2048	K21	2048	16	8	2048	1024
K6	1024	16	16	1024	1024	K22	1024	32	16	512	1024
K7	1024	16	16	512	1024	K23	2048	16	16	1024	2048
K8	512	8	16	512	512	K24	512	32	32	512	512
K9	2048	32	32	1024	2048	K25	2048	32	16	2048	1024
K10	1024	8	16	1024	512	K26	1024	16	32	512	1024
K11	1024	32	16	1024	1024	K27	1024	32	32	512	512
K12	2048	8	8	1024	2048	K28	1024	8	16	512	512
K13	512	32	16	512	512	K29	512	8	16	512	512
K14	1024	16	8	1024	1024	K30	1024	32	32	1024	1024
K15	1024	16	8	1024	1024	K31	2048	16	16	2048	1024
K16	2048	32	16	1024	1024	K32	2048	32	32	1024	1024

**Table 5:** The composition of the tested formulations (in weight ratio).

Formulation	Span 60	Cholesterol	NaCh	Lycopene	Water ad
F1	2	0.5	-	0.03	50
F2	2	0.5	0.2	0.03	50
F3	2	0.5	0.51	0.03	50
F4	2	0.5	0.82	0.03	50
Control *	**-**	**-**	**-**	0.03	50

* The control was aqueous lycopene suspension (600 μg/mL).

## Data Availability

Data is contained within the article and supplementary material.

## Supplementary Materials

The following supporting information can be downloaded at: https://www.mdpi.com/article/10.3390/ph15091043/s1, Table S1: The inhibition zone diameters of the free lycopene, its formulations, and the non-medicated formulations. Figure S1: A representative example for the results of disc diffusion method of lycopene (A), F2 formula (B), and (C) non-medicated F2 formula.
