# Supplementary material for: Bilosomes as Nanoplatform for Oral Delivery and Modulated In Vivo Antimicrobial Activity of Lycopene
Source: Pharmaceuticals (Basel). 2022 Aug 24;15(9):1043. doi: 10.3390/ph15091043 (PMC9505130; doi:10.3390/ph15091043)
Supplement: Supplementary file 1 [file pharmaceuticals-15-01043-s001.zip › pharmaceuticals-1847264-supplementary.pdf]

Table S1. The inhibition zone diameters of the free lycopene, its formulations, and the non-medicated formulations.

Isolate Code		Inhibition zone dimeters (mm)*							
	Free lycopene	F1 formula	F1 non – medicated **	F2 formula	F2 non – medicated **	F3 formula	F3 non – medicated **	F4 formula	F4 non – medicated **
K1	8.3±0.4	21±0.0	-	23±0.0	-	12.6±0.4	-	13±0.	-
K2	8±0.8	18±0.0	-	21.3±0.4	-	11±0.0	-	13.6±0.4	-
K3	9±0.0	19±0.0	-	26±0.0	-	9.3±0.4	-	10±0.0	-
K4	10.6±0.4	21.3±0.4	-	23.6±0.4	-	13.3±0.4	-	13.6±0.4	-
K5	10.3±0.4	22±0.0	-	25±0.0	-	10.3±0.4	-	11±0.0	-
K6	10.3±0.4	23.6±0.4	-	23±0.0	-	11.3±0.4	-	11±0.0	-
K7	7.3±0.4	26.6±0.4	-	27.3±0.4	-	11±0.0	-	11.6±0.4	-
K8	11.3±0.4	27.3±0.4	-	28±0.0	-	11.6±0.4	-	13±0.0	-
K9	8±0.0	23±0.0	-	25±0.0	-	9±0.0	-	10±0.0	-
K10	9±0.0	25±0.0	-	28±0.0	-	9±0.0	-	11±0.0	-
K11	8±0.0	23.6±0.4	-	26±0.0	-	13.6±0.4	-	14.3±0.4	-
K12	11±0.0	19±0.0	-	20.6±0.4	-	13.6±0.4	-	13.6±0.4	-
K13	12±0.0	20.6±0.4	-	26±0.0	-	13±0.0	-	14±0.0	-
K14	7.3±0.4	21±0.0	-	28±0.0	-	10±0.0	-	10±0.0	-
K15	8±0.0	26±0.0	-	26±0.0	-	9.6±0.4	-	8±0.8	-
K16	8±0.0	25±0.0	-	28±0.0	-	11±0.0	-	12±0.0	-
K17	9±0.0	23.6±0.4	-	26.6±0.4	-	9±0.0	-	11±0.0	-
K18	7±0.0	26±0.0	-	27±0.0	-	10.3±0.4	-	7.3±0.4	-
K19	11.3±0.4	23±0.0	-	24.3±0.4	-	12±0.0	-	13±0.0	-
K20	12±0.0	24.3±0.4	-	27±0.0	-	12±0.22	-	13.6±0.4	-
K21	11.3±0.4	26±0.0	-	27.3±0.4	-	11±0.0	-	11.6±0.4	-
K22	9.6±0.4	27±0.0	-	29±0.0	-	10.3±0.4	-	10±0.0	-
K23	10.6±0.4	27.3±0.4	-	28±0.0	-	12.3±04	-	13±0.0	-
K24	9±0.0	29±0.0	-	28±0.0	-	9±0.0	-	10.6±0.4	-
K25	8±0.0	23.6±0.4	-	25±0.0	-	9.6±0.4	-	12±0.0	-
K26	12±0.0	26±0.0	-	27.3±0.4	-	12.3±0.4	-	12±0.0	-
K27	10.3±0.4	27±0.0	-	29±0.0	-	11±0.0	-	11±0.8	-
K28	11±0.8	28.3±0.4	-	29±0.0	-	13.3±0.4	-	13±0.0	-
K29	11.6±0.4	23±0.0	-	23.6±0.4	-	12.6±0.4	-	12±0.0	-
K30	12.3±0.4	21.3±0.4	-	25±0.0	-	12.6±0.4	-	14±0.0	-
K31	7.6±0.4	19±0.0	-	20±0.0	-	10.3±0.4	-	11.6±0.4	-
K32	9±0.0	23.6±0.4	-	28.3±0.4	-	9.6±0.4	-	10.3±0.4	-

* The results are expressed as mean± standard deviation

** The non-medicated formulation didn't exhibit any inhibition zones around their disks.

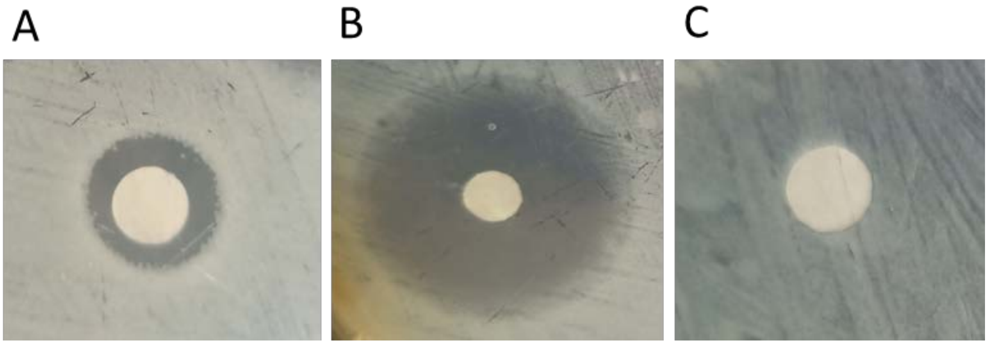

Figure S1. A representative example for the results of disc diffusion method of lycopene (A), F2 formula (B), and (C) non-medicated F2 formula.
